# The re-discovered *Maculinea rebeli* (Hirschke, 1904): Host ant usage, parasitoid and initial food plant around the type locality with taxonomical aspects (Lepidoptera, Lycaenidae)

**DOI:** 10.3897/zookeys.406.7124

**Published:** 2014-04-29

**Authors:** András Tartally, Anton Koschuh, Zoltán Varga

**Affiliations:** 1 Department of Evolutionary Zoology and Human Biology, University of Debrecen, Egyetem tér 1, H-4032 Debrecen, Hungary; 2 Steyrergasse 72/8 A-8010 Graz, Austria

**Keywords:** Styrian Alps, nominotypic *Maculinea rebeli*, *Myrmica sulcinodis*, *My. sabuleti*, *Gentianella rhaetica*, *Ichneumon* cf. *eumerus*

## Abstract

The taxonomy of the myrmecophilous *Maculinea alcon* group (Lepidoptera: Lycaenidae) is highly debated. The host-plant and host-ant usage of these butterflies have conventionally been important in their identification. *Maculinea ‘rebeli*’ has generally been considered to be the xerophilous form of *Ma. alcon* (*Ma. alcon* *X* hereafter) with *Gentiana cruciata* as initial food plant. However, the type locality and all other known sites of *Ma. rebeli* are found above the coniferous zone, and are well separated from the lower regions where *Ma. alcon* *X* sites are found. Furthermore, no food plant and host ant data for the nominotypic *Ma. rebeli* have yet been published. Our aim was therefore to identify the host ant(s) of *Ma. rebeli* around the type locality and compare this with the host ant usage of nearby *Ma. alcon* *X*. Nests of *Myrmica* spp. (Hymenoptera: Formicidae) close to the host plants were opened on one *Ma. alcon* *X* (host plant: *Gentiana cruciata*) and two *Ma. rebeli* (host plant: *Gentianella rhaetica*, first record, confirmed by oviposition and emerging larvae) sites just before the flying period, to find prepupal larvae and pupae. Three *Myrmica* species (*My. lobulicornis*, *My. ruginodis*, *My. sulcinodis*) were found on the two *Ma. rebeli* sites, which parasitized exclusively *My. sulcinodis* (22 individuals in 7 nests). On the *Ma. alcon* *X* site *Myrmica sabuleti* and *My. lonae* were found, with *My. sabuleti* the exclusive host (51 individuals in 10 nests). *Ichneumon* cf. *eumerus* parasitized both butterflies. The results highlight the differentiation of *Maculinea rebeli* from *Ma. alcon* *X*, from both conservation biological and ecological points of view. Thus, it should be concluded that *Ma. rebeli* does not simply represent an individual form of *Ma. alcon* but it can be considered as at least an ecological form adapted to high mountain conditions both in its initial food plant and host ant species. In addition, it should be emphasized that *Ma. alcon* *X* (= *Ma. rebeli* auct. nec Hirschke) cannot be synonymised with *Ma. rebeli* (Hirschke, 1904).

## Introduction

Although *Maculinea* van Eecke, 1915 (Lepidoptera, Lycaenidae) has been synonymised with *Phengaris* Doherty, 1890 (see: Fric et al. 2007, Pech et al. 2007, Kudrna and Fric 2013) we continue to use the well-established generic name here, since (i) the case is still undecided by the International Commission on Zoological Nomenclature (ICZN 2012) and strong arguments for the precedence of *Maculinea* over *Phengaris* have been made (Balletto et al. 2010, and Comments on this Case); and (ii) Ugelvig et al. (2011) have shown that *Maculinea* represents a monophyletic sub-clade and we agree with their conclusion: “*We recommend that the nomenclatural debate is delayed until irrefutable evidence is provided*”.

*Maculinea* (abbreviated as “*Ma.*” below) butterflies are endangered species (Munguira and Martín 1999, Maes et al. 2004, Settele et al. 2005) and their larvae are social parasites of *Myrmica* Latreille, 1804 (Hymenoptera: Formicidae; abbreviated as “*My.*” below) ant colonies (Thomas et al. 1989). Larvae of *Maculinea alcon* ([Denis & Schiffermüller], 1775) initially feed on the seeds of gentian plants for about a month and are later taken in and raised by *Myrmica* colonies (Thomas et al. 1989). They have a “cuckoo” strategy, in which they mimic the odour (Thomas and Settele 2004, Nash et al. 2008, Akino et al. 1999) and sound (Barbero et al. 2009) of the host ant species, and are therefore fed like an ant larva (Thomas and Elmes 1998), although they also sometimes prey directly on the ant brood (Tartally 2004). The caterpillars develop within the host ant nest during the autumn, winter and spring, and will then either pupate there, or continue development for an additional year (Schönrogge et al. 2000, Witek et al. 2006).

There are regional and local differences between the host ant usage of different populations (e.g. Elmes et al. 1994, Als et al. 2002, Höttinger et al. 2003, Steiner et al. 2003, Sielezniew and Stankiewicz 2004, Stankiewicz et al. 2005, Tartally et al. 2008, Witek et al. 2008, Tartally et al. 2013). Knowledge of the local host ant species is vital to understand the biogeography, conservation and evolutionary biology of *Maculinea* spp. (Settele et al. 2005, Settele and Kühn 2009), and may also have some taxonomic implications. Furthermore, the host ant and host plant usage of *Maculinea alcon* has been thought to be important for taxonomic separation (Thomas et al. 1989) of the hygrophilous (‘*Maculinea alcon* *H*’ below) and the xerophilous (‘*Maculinea alcon* *X*’) form of *Maculinea alcon*, although recent studies have been unable to show any consistent genetic separation between these two butterflies (Als et al. 2004, Bereczki et al. 2005, Ugelvig et al. 2011). Despite this, *Maculinea alcon* *X* is still generally (and erroneously, see Kudrna and Fric 2013) referred to as ‘*Maculinea rebeli* (Hirschke, 1904)’ (e.g. Elmes et al. 1998, Steiner et al. 2003, Stankiewicz et al. 2005, Tartally et al. 2008). Some Balkan, Caucasian and Central Asian mountainous populations of *Maculinea alcon* have also been referred to as *Maculinea* ‘*rebeli*’ (Kolev 2002, Savela 2014, Tuzov 2000).

However, a recent paper (Habeler 2008) called our attention to the fact that *Maculinea alcon* *X* (= *Maculinea rebeli* auctorum nec Hirschke, 1904) is not likely synonymous with *Maculinea rebeli* described by Hirschke (1905) from the type locality, based on external morphological features and the different habitat types in which they occur. It has now been demonstrated that *Maculinea rebeli* auct. does not represent a distinct species, but rather the xerophilous form of *Maculinea alcon* (*Maculinea alcon* *X*). It feeds initially typically on *Gentiana cruciata* (Bereczki et al. 2005, Pecsenye et al. 2007, Sielezniew et al. 2012) then using mainly *Myrmica schencki* Viereck, 1903, *Myrmica sabuleti* Meinert, 1861 and some other available *Myrmica* species (e.g. Elmes et al. 1998, Meyer-Hozak 2000, Steiner et al. 2003, Stankiewicz et al. 2005, Tartally et al. 2008). However, following the paper of Habeler (2008), we realised that no reliable host ant and initial food plant data have been published for the nominotypic *Maculinea rebeli* (called simply *Maculinea rebeli* hereafter).

The main reason for our study is that the type locality and the other suggested sites of *Maculinea rebeli* are above the coniferous zone and well-separated by spruce forests (see Figure 7 in Habeler 2008) from the lower regions where *Maculinea alcon* *X* occurs in grasslands (mostly extensive pastures) with *Gentiana cruciata*. Furthermore, from our personal observations (see Materials and Methods), *Maculinea rebeli* uses *Gentianella rhaetica* (A. Kern. & Jos. Kern.) Á. Löve & D. Löve rather than *Gentiana cruciata* at higher altitudes, as the latter plant is not available in that zone (see the questions about host plant usage of Kudrna and Fric 2013). Similarly, we reasoned that the available *Myrmica* species are likely to be different for *Maculinea rebeli* and *Maculinea alcon* *X* within the same region because of changes in available niches with increasing altitude (Elmes et al. 1998, Glaser 2006). It is therefore important to know the host ant use and specificity of *Maculinea rebeli* and *Maculinea alcon* *X* around the type locality of *Maculinea rebeli* to help in answering the question of whether these two butterflies should be treated as the same or different forms or species.

## Methods

**Figure 1–4.**
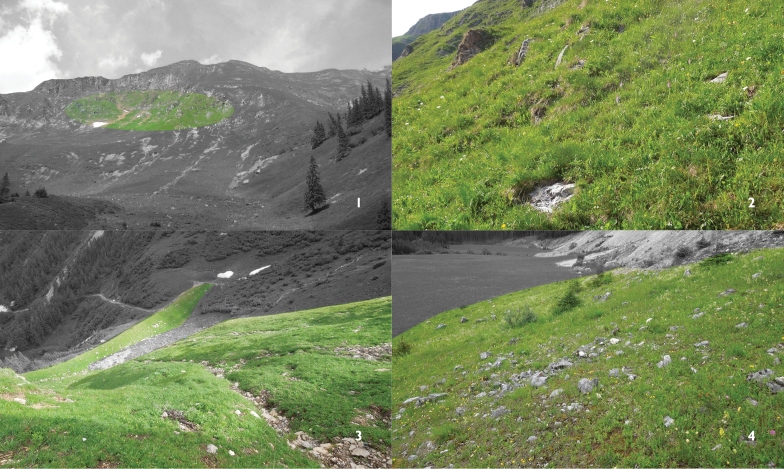
**1–2** *Maculinea rebeli* site at Zeiritz above the coniferous zone (1750 m) **3** *Maculinea rebeli* site at Präbichl above the coniferous zone (1600 m) **4** *Maculinea alcon* *X* site at Sankt Ilgen within the coniferous zone (photo: AK).

**Figure 5–7.**
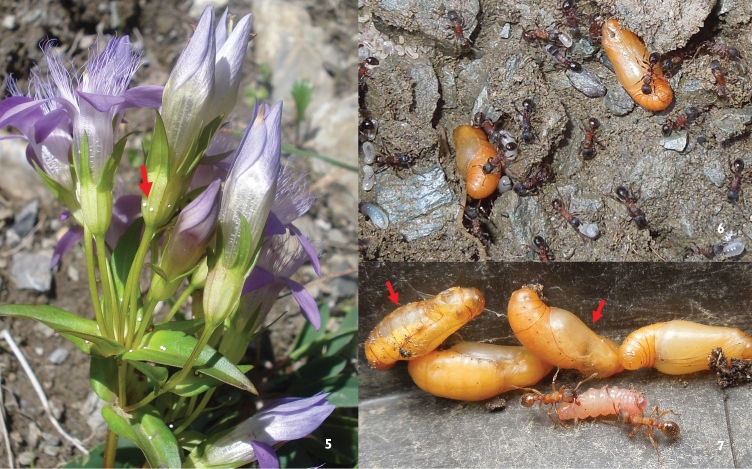
**5** *Maculinea rebeli* eggs on *Gentianella rhaetica* at Präbichl **6** *Maculinea rebeli* pupae under a stone in a *Myrmica lobulicornis* nest at Zeiritz **7** *Maculinea alcon* *X* pupae and a small larva found in *Myrmica sabuleti* nest at Sankt Ilgen, arrows sign infections with *Ichneumon* cf. *eumerus* (photo: AK).

**Figure 8–9.**
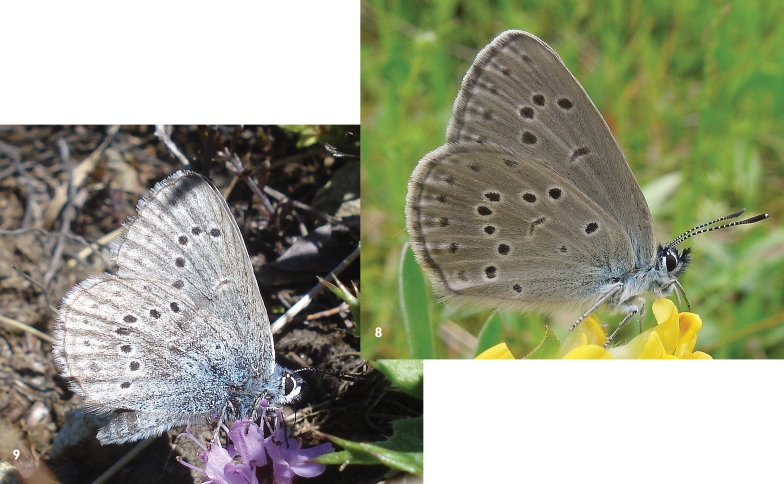
**8** *Maculinea alcon* *X* (Hochschwab area) **9** *Maculinea rebeli* (Hochschwab area) (photo: AK).

Three sites within the Hochschwab area (Styrian Alps, Austria; Figs 1–4) were visited between the 22^nd^ and 24^th^ June 2012 (just before the flying period). Two *Maculinea rebeli* habitats above the coniferous zone were investigated, one at Zeiritz (47.486N, 14.723E, ca 1750 m a.s.l.) and another at Präbichl (47.512N, 14.938E, ca 1600 m a.s.l.). Both sites are sparsely covered by calcareous alpine *Sesleria varia* (Jacq.) Wettst.-*Carex sempervirens* Vill. grassland (Seslerio-Semperviretum) vegetation on stony and sunny slopes, with bare limestone rocks (Figs 1–3, compare with Figs 8–10 in Habeler 2008). We regularly found eggs on *Gentianella rhaetica* (Fig. 5; det. K. Zernig, Universalmuseum Joanneum; it belongs to the *Gentianella germanica* agg.: Greimler et al. 2011) on both sites, and caterpillars readily developed on and emerged from this plant (AK and AT, pers. observ.). The third site is a calcareous grazed meadow (Fig. 4) within the coniferous zone (Sankt Ilgen: 47.562N, 15.153E, ca 810 m a.s.l.) where *Maculinea alcon* *X* uses *Gentiana cruciata* as initial host plant (an adjacent similar site for *Maculinea alcon* *X* has already been described by Bereczki et al. 2005 and Pecsenye et al. 2007).

The host ant specificity of the *Maculinea alcon* *X* population at Sankt Ilgen was checked by searching for *Myrmica* spp. nests within 2 m of *Gentiana cruciata* plants (see details: Tartally et al. 2008). However, this method was not possible in the case of the two *Maculinea rebeli* sites (at Präbichl and Zeiritz) as *Gentianella rhaetica* plants are rather small before the flying period of *Maculinea rebeli*, and were only rarely visible. We instead worked within patches where *Gentianella rhaetica* plants were found in the previous year (although it should be noted that we were often not sure of the actual presence of plants in 2012, as *Gentianella rhaetica* is biannual). When a *Myrmica* nest was found, it was opened as carefully as possible to search for *Maculinea* larvae and pupae. Five to ten workers were collected from each ant nest and preserved in 75% ethanol for identification in the laboratory (following keys in Seifert 1988, Radchenko and Elmes 2010, Czechowski et al. 2012).

Host ant specificity was calculated based on the number of fully grown butterfly larvae, pupae and exuvia found (i.e., not including small larvae: see results) in two ways (see details: Tartally et al. 2008): p1 is the 2-tailed probability from a Fisher exact test of heterogeneity in infection of host ant nests (as implemented at http://www.quantitativeskills.com/sisa/), and p2 is the probability from a randomization test of ant nests between species (using the program MacSamp; Nash 2014). We should emphasize that our data on the host ant specificity of *Maculinea rebeli* and *Maculinea alcon* *X* should be compared cautiously, as our certainty of the presence of host plants differed for *Maculinea rebeli* and *Maculinea alcon* *X* (see above). Furthermore, excavating complete nests of *Myrmica* spp. was relatively easy in the deep humus of the *Maculinea alcon* *X* site, but was much more difficult in the rocky *Maculinea rebeli* sites.

## Results

Altogether 53 *Myrmica* nests were found on the three sites (Table 1), consisting of 52 nests of five *Myrmica* species and one empty (presumably recently vacated) but *Maculinea*-infested nest which was similar in appearance to nearby *Myrmica sabuleti* nests. Only nests of *Myrmica sabuleti* and of the closely related (Radchenko and Elmes 2010) *Myrmica lonae* Finzi, 1926 were found at the *Maculinea alcon* *X* site (Sankt Ilgen), whereas the *Myrmica* composition of the two *Maculinea rebeli* sites was totally different from this: *Myrmica ruginodis* Nylander, 1846 and *Myrmica sulcinodis* Nylander, 1846 were found at both sites, plus *Myrmica lobulicornis* Nylander, 1857 at Präbichl.

**Table 1.** The recorded *Myrmica* spp. nests and host ant usage of *Maculinea rebeli* (at Präbichl and Zeiritz), *Maculinea alcon* *X* (at Sankt Ilgen) and *Ichneumon* cf. *eumerus* according to the number of large larvae, pupae and exuvia (= “grown *Maculinea*”) and according to these together with the small larvae (= ”*Maculinea* in total”). Two measures of host specificity are given: P1 is the 2-tailed probability from the Fisher exact test of heterogeneity in infection of host ant nests and P2 is the probability from a randomization test of ant nests between species (see Materials and Methods).

Site	No. *Myrmica* nests	Nests Infested with grown *Maculinea*	No. grown *Maculinea* (range, P1, P2)	Nests Infested with *Maculinea* in total	No. *Maculinea* in total (range, P1, P2)	No. nests Parasitized with *Ichneumon* cf. *eumerus*	No. *Ichneumon* cf. *eumerus* (range, P1, P2)
Präbichl	lobulicornis 3						
(*Maculinea rebeli*)	ruginodis 1						
	sulcinodis 13	4	15	6	25	2	8
			(2–7, 0.64, 0.62)		(2–7, 0.34, 0.39)		(2–6, 0.43, 0.86)
Zeiritz	ruginodis 8						
(*Maculinea rebeli*)	sulcinodis 13	3	7	3	8	1	1
			(2–4, 0.26, 0.35)		(1–3, 0.26, 0.25)		(1–1, 1.00, 1.00)
Sankt Ilgen	lonae 1						
(*Maculinea alcon* *X*)	sabuleti 13	10	51	10	53	9	28
			(1–11, 0.26, 0.50)		(1–11, 0.26, 0.50)		(1–8, 0.36, 0.64)
	empty nest 1	1	9	1	9	1	8

Nine nests of *Myrmica sulcinodis* were infested with 33 *Maculinea rebeli* larvae, pupae and exuvia at Präbichl and Zeiritz in total, of which 11 caterpillars were so small as to be two-year developing larva after their first winter. There were 15 pupae in total on these two sites, nine of which (60%) proved to be infected with *Ichneumon* cf. *eumerus* Wesmael, 1857.

Ten nests of *Myrmica sabuleti* were infested at Sankt Ilgen with 53 *Maculinea alcon* *X* larvae, pupae and exuvia, but only two caterpillars were so small as to be two-year developing larvae. There were nine “orphaned” *Maculinea alcon* *X* pupae in the empty nest, which were dirty and showed visible signs of external mould. There were 36 pupae in total, 28 of which (77.8%) were infected with *Ichneumon* cf. *eumerus*.

## Discussion

Based on the character of the habitat, the figures published by Habeler (2008) and photographs of *Maculinea rebeli* imagos taken by AK (Figs 8–9), we are confident that we have re-discovered two populations that correspond to the description of Hirschke (1905). Our data confirm that *Maculinea rebeli* is (at least) an ecological form found at high altitudes, and with host ant and initial food plant distinct from the adjacent populations of *Maculinea alcon* *X* (*Maculinea rebeli* auct. nec Hirschke). It is unlikely to be a mere individual form characterised “*by the presence of a band of whitish or silver-greyish spots in the blue ground colour in the submarginal band adjacent to the marginal black line on the upper side of wings, particularly well pronounced on the hind wings and especially in the* ♀” (see Kudrna and Fric 2013 page 117, which is a misinterpretation of the original German text of Hirschke (1905): 109–110: „*Am Innenwinkel der Vorderflügel zeigt sich in Zelle 2 und 3 eine auffallende Aufhellung, welche sich auf die Hinterflügel fortsetzt und gegen den Innenwinkel derselben sogar die Form einer Antemarginalbinde gewinnt*.”). Furthermore, the specimens figured by Habeler (2008) also clearly show this character of *Maculinea rebeli* but they are simply darker than the type series of Hirschke (1905) due to the circumstance that they were captured quite recently (1978–2005) while the type series was collected in 1904.

To avoid further confusion, we argue that the true *Maculinea rebeli* occurs in the Hochschwab area above the coniferous zone (between c.a. 1500 and 2000 m) on slightly covered stony and sunny slopes with uncovered chalk rocks, where *Gentianella rhaetica* is the only recorded initial host plant, and *Myrmica sulcinodis* is the only recorded host ant species so far. We still need to confirm the observation of Habeler (2008) that these high altitude habitats are usually small, fragmented, and often located on steep and inaccessible (see Fig. 1) slopes, but it does seem that these populations have remained unknown to (or neglected by) most authors (see Kudrna and Fric 2013).

As far as we know, our observations are the first data on the host ant specificity of *Maculinea rebeli* around the type locality, and hence, we maintain, of the host ant specificity of *Maculinea rebeli*. Earlier data about the host ant specificity of *Maculinea* ‘*rebeli*’ (e.g. Elmes et al. 1998, Meyer-Hozak 2000, Steiner et al. 2003, Stankiewicz et al. 2005, Tartally et al. 2008) should instead be considered as data about the host ant specificity of *Maculinea alcon* *X* (see Introduction and Habeler 2008). While *Maculinea rebeli* was found exclusively in *Myrmica sulcinodis* nests at Zeiritz and Präbichl, the works just cited mention *Maculinea alcon* *X* generally from nests of *Myrmica schencki* or *Myrmica sabuleti* and from some other locally available *Myrmica* species (*Myrmica lonae*; *Myrmica scabrinodis* Nylander, 1846; *Myrmica specioides* Bondroit, 1918; *Myrmica rugulosa* Nylander, 1849; and *Myrmica ruginodis*). However, we cannot rule out the discovery of other host ants of *Maculinea rebeli* in the Hochschwab region, following more intensive work. H.C. Wagner (see Kudrna and Fric 2013) also found *Myrmica sulcinodis*, *Myrmica ruginodis* and *Myrmica lobulicornis* as available *Myrmica* species on *Maculinea rebeli* sites, plus one nest of *Myrmica scabrinodis*, and also suggested *Myrmica lobicornis* Nylander, 1846 as a potentially available but unrecorded species. Jutzeler (1989) has previously recorded *Myrmica sulcinodis* interacting with a full-grown larva of *Maculinea* ‘*rebeli*’ in the Swiss alps, although it was unclear whether this was a host nest or opportunistic predation (see also: Kudrna and Fric 2013). Although he did not record the altitude of this observation, it is notable that he also observed oviposition by *Maculinea* ‘*rebeli*’ on *Gentianella germanica* (Willd.) E.F.Warb. on the same site (Jutzeler 1988). This region is distant and isolated by high mountains from the type locality of *Maculinea rebeli*. Genetic studies and more knowledge about the host ant specificity of this Swiss population would be necessary to decide whether it is really *Maculinea rebeli* (and e.g. not *Maculinea alcon* *X*) and if its main host ant is really *Myrmica sulcinodis*. Here it should be noted that specimens, phenotypically completely identical with the type specimens of *Maculinea rebeli*, have also been collected from some high mountain habitats of Switzerland (Pontresina, 21. 07. 1907, leg. F.W. Dadd, coll. Zoological State Collection, Munich, checked by ZV).

Our results for *Maculinea alcon* *X*, showing *Myrmica sabuleti* as host, are in agreement with previous studies (e.g. Meyer-Hozak 2000, Tartally et al. 2008) that *Myrmica sabuleti* is one of the most important host ants of this butterfly, and which has been already identified as the main host in other Austrian sites by Steiner et al. (2003).

The presence of *Ichneumon* cf. *eumerus* in *Maculinea* pupae appears to be a good indication that a given ant species is a suitable host ant of *Maculinea* butterflies (Thomas et al. 2002), so the relatively high infestation rate by the parasitoid is consistent with *Myrmica sabuleti* being a suitable host of *Maculinea alcon* *X*, and with *Myrmica sulcinodis* being a suitable host of *Maculinea rebeli* in the sites studied here.

The one empty *Myrmica* nest found to be infested with *Maculinea alcon* *X* confirms that colonies of various *Myrmica* species can migrate (Radchenko and Elmes 2010). Therefore it could potentially happen that a host *Myrmica* colony moves out and leaves the *Maculinea* pupae in the nest and another *Myrmica* species occupies the empty nest, complete with *Maculinea* pupae, leading to an erroneously recorded ‘secondary host ant’ (Thomas et al. 2005).

It is almost certain that *Manica rubida* Latreille, 1802 is not available as a potential host for the populations studied here, despite the presence of this ant in this region. *Manica rubida* is closely related genetically (Jansen and Savolainen 2010), and similar chemically (Errard et al. 2006), to *Myrmica* spp. and shows potential suitability as a host of the *Maculinea alcon* group in the laboratory (Tartally 2004). This ant is common in the nearby sites of both butterflies, typically in the border of spruce forests, but was not found in the patches with *Gentiana cruciata* or *Gentianella rhaetica*. It also should be noted that despite the common presence of other gentian species (e.g. *Gentiana asclepiadea* L., see also: Kudrna and Fric 2013) in this region, *Maculinea* eggs were never observed on them.

The unusual host ant use of *Maculinea rebeli* in the Alpine sites could be constrained by the distinctive composition of the *Myrmica* community found there. Although *Maculinea* populations can adapt to locally available *Myrmica* species (e.g. Nash et al. 2008, Tartally 2005, Tartally et al. 2013), the unique combination of host ant and host plant usage of these *Maculinea rebeli* populations means that the nominotypic *Maculinea rebeli* should be treated as a distinct Conservationally Significant Unit (see Maes et al. 2004), separated biologically and ecologically from both *Maculinea alcon* *X* and *Maculinea alcon* *H*. Further studies on the host ant and host plant specificity of *Maculinea rebeli* and genetic comparison of these butterflies is necessary to qualify the degree of ecological and genetic relationships within the *Maculinea alcon* group, and also to draw well-supported taxonomical conclusions on the status of the nominotypical *Maculinea rebeli* versus *Maculinea rebeli* auct. (*Maculinea alcon* *X*).

## Conclusion

It should be concluded that *Maculinea rebeli* does not simply represent an individual form of *Maculinea alcon* (as considered by Kudrna and Fric 2013) but it should be considered as at least an ecological form adapted to high mountain conditions both in its initial food plant and host ant species. In addition, it should be emphasized that *Maculinea alcon* *X* (= *Maculinea rebeli* auct. nec Hirschke) cannot be synonymised with *Maculinea rebeli* (Hirschke, 1904).

**Figure 10.**
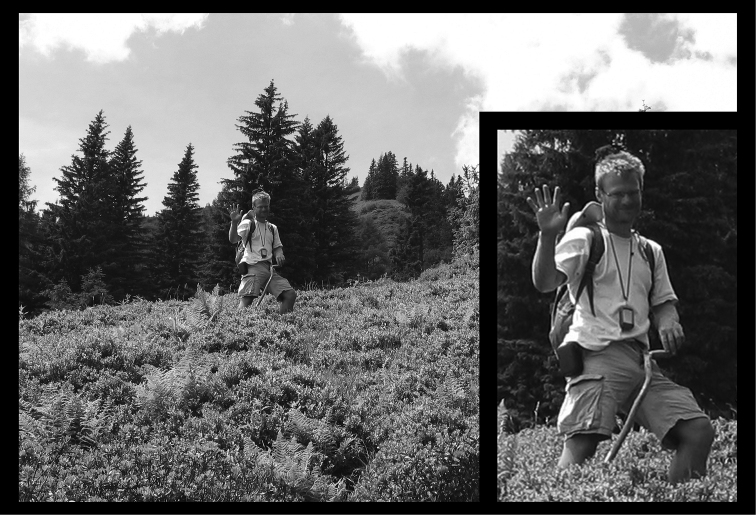
Anton Koschuh on his way to Zeiritz (23.06.2012) (photo: AT).
